# Tape Worm

**Published:** 1855-08

**Authors:** 


*Tape Worm.*—We continue to meet with occasional reports of cases cured by pumpkin seed. The efficacy of this remedy has now been sufficiently proved to give it a preference over all others. Dr. Monkur, of Baltimore, gave it to a patient as follows: Bruise six ounces of the seed in a mortar, without removing the shells, and add water sufficient to give by expression, one pint of liquid. One half this is taken at a dose, followed in two hours by two tablespoonfuls of castor oil. In seven or eight hours these doses are repeated. His patient discharged large quantities of tape worm, and one entire worm, measuring seventeen feet. He considers it important to use fresh and sound seeds, and thinks those grown in the West Indies, preferable. The pumpkin of the Southern States, differing somewhat from the Northern pumpkin, is probably the same as that of Jamaica, where this remedy first came into notice.—*Memphis Med. Recorder.*

